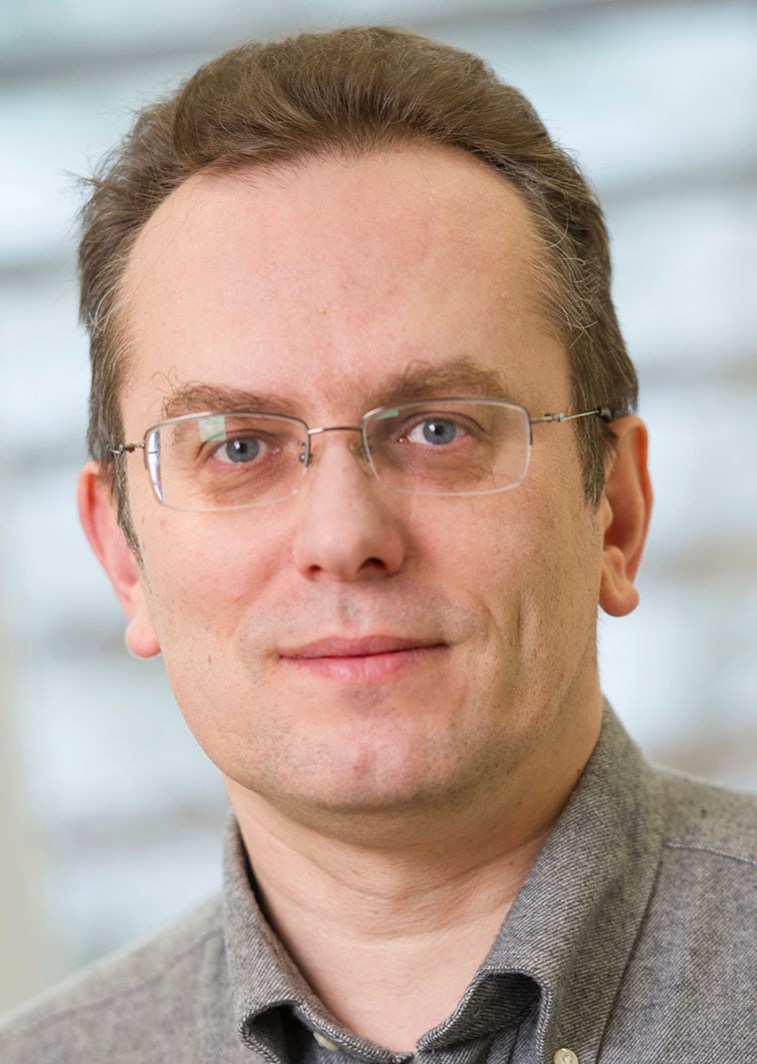# A Survey of Multi‐task Learning Methods in Chemoinformatics

**DOI:** 10.1002/minf.201800108

**Published:** 2018-11-28

**Authors:** Sergey Sosnin, Mariia Vashurina, Michael Withnall, Pavel Karpov, Maxim Fedorov, Igor V. Tetko

**Affiliations:** ^1^ Center for Computational and Data-Intensive Science and Engineering Skolkovo Institute of Science and Technology Skolkovo Innovation Center Moscow 143026 Russia; ^2^ Helmholtz Zentrum München – German Research Center for Environmental Health (GmbH) Institute of Structural Biology Ingolstädter Landstraße 1 D-85764 Neuherberg Germany; ^3^ University of Strathclyde Department of Physics John Anderson Building, 107 Rottenrow East G40NG Glasgow United Kingdom; ^4^ BIGCHEM GmbH Ingolstädter Landstraße 1, b. 60w D-85764 Neuherberg Germany

**Keywords:** Multi-task learning, transfer learning, neural networks

## Abstract

Despite the increasing volume of available data, the proportion of experimentally measured data remains small compared to the virtual chemical space of possible chemical structures. Therefore, there is a strong interest in simultaneously predicting different ADMET and biological properties of molecules, which are frequently strongly correlated with one another. Such joint data analyses can increase the accuracy of models by exploiting their common representation and identifying common features between individual properties. In this work we review the recent developments in multi‐learning approaches as well as cover the freely available tools and packages that can be used to perform such studies.

## 1 Introduction

Nowadays, the volume of data that can be generated and processed when modelling tasks has increased dramatically.^1^ Machine Learning (ML) techniques, notably Deep Neural Networks (DNNs)^2^ are becoming indispensable as a tool for managing and using these vast amounts of generated and measured data effectively. However, data measurement is still a difficult and time‐consuming task, and there is a strong interest in how to make the best use of all available data. Biological data, such as ADMETox properties, are highly interrelated. For example, the lipophilicity of compounds is, in one way or another, very important for the majority of these properties. Thus learning several ADMETox properties simultaneously can result in better models. Moreover, some types of data produced with different methods can have different experimental accuracy and/or refer to related but not identical properties. For example, kinetic water solubility is the concentration of a compound in solution at the time when an induced precipitate first appears. This type of solubility can be easily automatized for use in High Throughput Screening (HTS) settings and is actively used in industry due to this. The more biologically relevant solubility is thermodynamic solubility, which is the concentration of a compound in a saturated solution when excess solid is present, and solution and solid are at equilibrium.^3^ The co‐modelling of both types of solubility simultaneously could potentially develop better models for each of them. This can be achieved with the help of multi‐task learning,^4^ which can be applied to an arbitrary combination of regression and classification tasks (so called heterogeneous multi‐tasks).

These multi‐learning approaches belong to so‐called transfer learning,^5^ a technique where knowledge gained in one or several (source) tasks is used to improve the target task. The transfer learning approaches differ with respect to whether the source and/or target tasks have labelled data. Thus, they can be classified as semi‐supervised or “self‐taught” learning (no labelled data in the source domain), transductive learning (labelled data are only in the source domain), unsupervised transfer learning (no labelled data are available)^5^ as well as methods which use labelled data for both source and target tasks, which include multi‐learning approaches.

**Figure 1:**
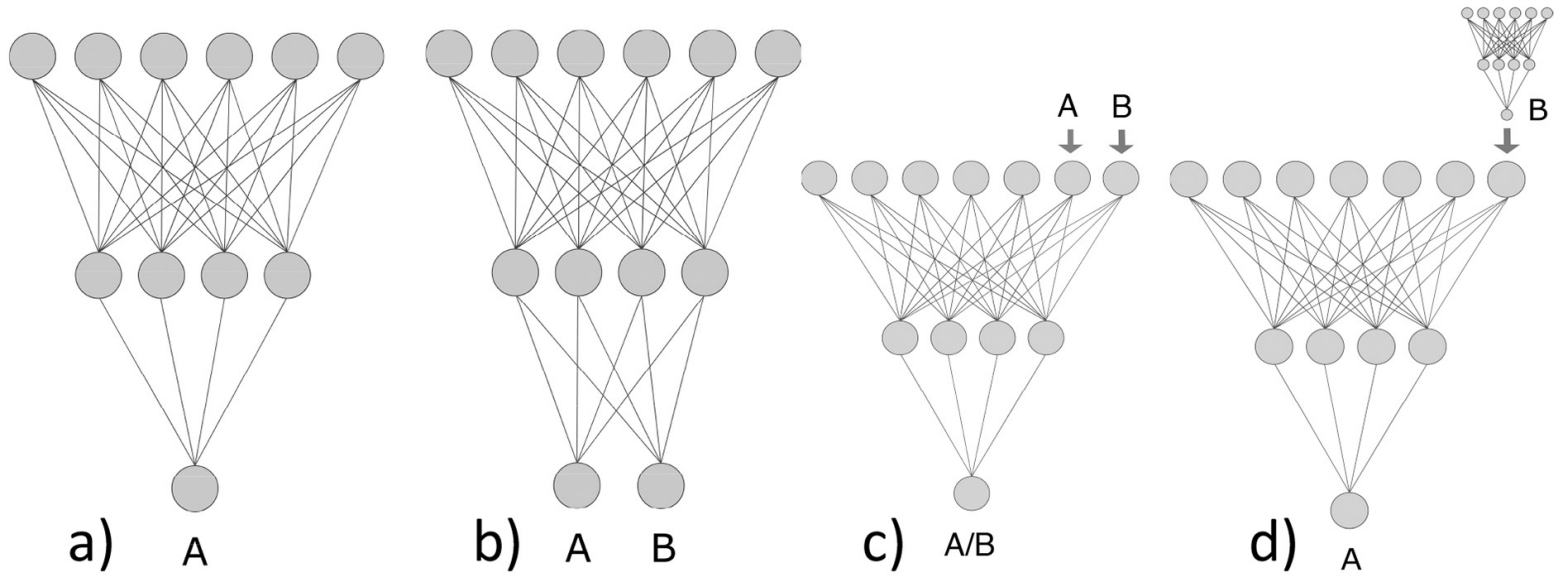
a) Single Task learning; b) Multi‐task learning; c) Multi‐task learning by property encoding as descriptors; d) Feature net. Adapted with permission from ref. [8]. Copyright (2009) American Chemical Society.

The ability to infer relevant knowledge is very important for intelligence. For example, humans, who can draw on vast amounts of previously‐learned information, can be trained on a new task with a relatively tiny number of examples. In contrast, traditional machine learning algorithms, which usually learn from scratch, and require large example sets to do so. Therefore, there is active development and interest in machine learning to design new methods having the same speed and accuracy as humans. Early examples of such types of learning have been successfully reported since the mid‐1990s, e. g. the use of neural network weights trained with one task as a starting point for new ones to increase the development speed and the accuracy of models.^4^ A Library model of Associative Neural Networks^6^ is another example, which applied on‐the fly correction of predictions for new data by using the errors of the nearest neighbours of the target sample.^7^ Transfer of information was also done by developing models for individual properties, and then using those model predictions as additional descriptors for the target property, known as the feature net approach.^8^ In the case that the target and source properties are very similar or identical (e. g., measured for different species or at different conditions), one can encode different targets by using additional descriptors (e. g., conditions of experiments) and model all properties simultaneously. Figure 1 schematically illustrates single task as well as several multitask modelling approaches using an example of neural networks. Some of these approaches, such as the feature net, use sequentially‐ordered learning.

In our review we will cover new developments in the field, which have appeared during the recent years. Also, we will mainly focus on the methods where the analysed properties are simultaneously modelled within a single model, which corresponds to Figure 1b.

Multi‐task Learning (MTL) is a technique which aims improve ML efficacy by simultaneously co‐modelling multiple properties within a single model. A lot of developments in this field were done in in 1990s by Rich Caruana,^4^ who investigated how to improve related task performance by leveraging domain‐specific information, and inductively transferring it between the tasks. In comparison to the other transfer learning approaches, which use labelled data for both source and target tasks, the aim of MTL is to improve the performance of all tasks with no task prioritised.

MTL trains tasks in parallel, sharing their representation internally. As a result, the training data from the extra tasks serve as an inductive bias, acting in effect as constraints for the others, improving general accuracy and the speed of learning. Caruana noted mechanisms by which MTL may show improvement over Single Task Learning (STL) to be a) amplification of statistical data; b) attention focusing (finding a better signal in noisy data); c) eavesdropping (learning “hints” from simpler tasks); d) representation bias and feature selection and e) regularisation (less overfitting).^4^

As MTL implies sharing information between all tasks, it is possible to define three main types of MTL based on the type of data sharing: feature, instance and parameter‐based.^9^ Feature‐based MTL models learn a common feature representation among all the tasks by assuming that such a representation can increase the performance of the algorithm vs. single‐tasks. Parameter‐based approaches explore the similarity between target properties and include task clustering, learning of task relationships, as well as multilevel hierarchical approaches. Instance‐based MTL identifies individual data within a task, which can be effectively used in other tasks for information sharing.^10^ However, we did not find applications for the latter in chemoinformatics and thus will not cover them in our review. Let us consider some examples of the other two MTL approaches and their combination.

## 2 Feature Based Approaches

Neural networks are the primary platform for multi‐learning. Rich Caruana was one of the first to develop multi‐task learning using backpropagated neural networks. He found out that four separate neural networks performing only one task can be reduced to one network with multiple outputs that performs the tasks simultaneously. As a result, he created a multi‐task neural network able to perform parallel learning. One should also mention the earlier work of Suddarth and Kergosien,^11^ who used an additional layer to inject rule hints and to guide the neural network as to what should be learned.

The network forms a set of features on the hidden layer(s), which can fit several tasks simultaneously. Moreover, the activation patterns of neurons in neural networks with several hidden layers contribute to the formation of features, which are known to be important for the analysed type of properties, e. g. toxicophores for the prediction of toxicological end‐points.^12^

One of the first successful applications of MTL in chemoinformatics was done by Varnek et al.,^8^ who demonstrated that learning several tissue/air partitioning coefficients by using Associative Neural Networks provided models with statistically‐significantly higher accuracy compared to the respective single task models. The neural network models analysed by Varnek et al. were examples of so‐called “shallow” neural networks since they included only one hidden layer. The appearance of new training algorithms and in particular GPU‐accelerated computing has brought about the rise of Deep Neural Networks,^2^ which incorporate multiple hidden layers with much larger numbers of neurons. This greater flexibility of DNN networks allows them to learn more complex relationships and patterns in the data.

Regarding multi‐learning one can distinguish two primary architectures with respect to the sharing of parameters: hard and soft. “Hard” parameter sharing is similar to that of shallow neural networks and implies the sharing of hidden layers between all tasks, except some task‐specific output layers. “Soft” parameter sharing gives each task its own model with its own parameters, where these model parameters have a regularized distance to facilitate the sharing of learning.^13^ Soft parameter sharing has not yet received sufficient attention in chemoinformatics and will be briefly outlined in the section “Simultaneous Feature and Task similarity learning”.

J. Ma et al.^14^ performed several experiments on STL and MTL neural networks. They found out that in some cases multi‐task learning deep neural networks (MTL DNN) are better than single task learning deep neural networks (STL DNNs). The authors suggested that better performance of MTL DNN is based mainly on the size of data sets: MTL DNNs are useful for small and mixed (small and large) datasets and STL DNNs are good for large data sets.

Multi‐task learning provided the best model according to the ROC AUC (Receiver Operator Characteristic Area Under Curve) metric for the Tox21 challenge.^12^ The authors showed that such networks learned on their hidden layers chemical features resembling toxicophores identified by human experts. The networks used these features to classify active and inactive (toxic and nontoxic) compounds. It is also of note that the second best approach was based on “shallow” STL associative neural networks.^15^

In another comprehensive study the authors compared the performance of MTL and STL approaches in predicting the toxicity of chemical compounds from the Registry of Toxic Effects of Chemical Substances (RTECT) database totalling 29 toxicity end‐points and more than 120 k measurements.^16^ MTL significantly outperformed STL thus showing the utility of this approach to model complex *in vivo* endpoints.

Xu et al.^17^ investigated why an MTL DNN can outperform separate STL DNNs and under what scenarios the multi‐task approach is advantageous. The result of this study lead to two main findings regarding the efficacy of multi‐task deep neural networks:

- Similar molecules modelling correlated properties will boost the predictive performance of the DNN, and likewise uncorrelated properties will degrade performance.
- Structurally dissimilar molecules have no influence on the predictive performance of the MTL DNN, regardless of whether or not tasks are correlated.

Their conclusions are important for the identification of strategies for designing datasets for MTL learning.

MTL can be used to simultaneously learn both regression and classification in one model, as was demonstrated by Xu et al.^18^ for the prediction of acute oral toxicity. The authors used convolutional neural networks and reported that their model provided higher accuracy compared to conventional methods.

Human cytochrome P450 inhibition for 5 kinases were predicted using a pre‐trained autoencoder‐based DNN.^19^ On the pre‐training stage, the first layers were trained to reconstruct the original input layer on the whole database. The authors proved that an autoencoder‐based DNN can achieve better quality than other popular methods of machine learning for cytochrome P450 inhibition prediction, and a multi‐target DNN approach can significantly outperform single‐target DNNs. The flexibility of neural networks makes it possible to use them not only with descriptors derived from chemical structures in the traditional way, but also apply them to directly analyse chemical structures represented as SMILES or chemical graphs. We will review several approaches in the “Implementations of multi‐learning approaches” section below.

**Multi‐task feature learning for sparse data using other methods**. The problem of feature‐selection has an exact mathematical formulation and an analytical solution for linear methods. For example, Varnek et al.^8^ compared the performance of neural networks with Partial Least Squares (PLS). PLS could also provide multi‐task learning by identifying common internal representations, so called latent variables, for several analysed properties simultaneously. In addition to the PLS method, there are other approaches for identifying sparse features or to perform multi‐feature selection as comprehensively analysed in a recent review.^9^ These methods can be used directly with linear or kernel methods, or to provide features for training other methods.

One such method is Macau.^20^ It is based on Bayesian Probabilistic Matrix Factorisation (BPMF). After BPMF was used to win the Netflix prize for predicting film recommendation, the interest in this method notably increased. One of the problems during multi‐learning are missing values; frequently not all measurements are available for all targets. For some other tasks the matrix of responses can be extremely sparse, e. g. only 1.2 % of all users‐combinations were available for the Netflix competition. Some methods, such as neural networks, can naturally work with missing values by ignoring the error contribution from missing values when calculating the loss for backpropagation. The BPMF allows imputing missing values in the matrix thus enabling the application of standard techniques, such as singular value decomposition and principal component analysis. In contrast to classical algorithms of matrix factorization, Macau is able to handle side relations i. e. fingerprints of chemical compounds or phylogenetic distance between protein targets. Another useful feature of Macau is the ability to work with multi‐dimensional data and perform tensor decomposition. The capacity to deal with multi‐dimensional biological sparse data was studied by de Vega et al.,^21^ who applied this technique to inhibition activities of 15073 compounds for 346 targets extracted from ChEMBL. The authors showed that Macau provided performance similar to that of neural networks methods but did not require GPU‐accelerated computing.

## 3 Task Learning Approaches

Task learning explores task relationships to better learn common parameters of models as overviewed below.

**Metric‐learning algorithms**. k‐Nearest Neighbour approaches provide predictions for new samples based on their nearest neighbours. Usually, it uses a Mahalanobis distance, which is defined as:

(1) $$d_{M}\left(x_{i}{,x}_{j}\right)=\sqrt{{\left(x_{i}-x_{j}\right)}^{T}M\left(x_{i}-x_{j}\right)}$$

where and x_i_ and x_j_ are two samples and M is a matrix, which acts as a global linear transformation of the input space. The M matrix is thus an optimizable parameter of the method. The most straightforward way is to use the same metric to model all tasks simultaneously. However, better performance can be expected by using different matrices, which are optimised to each individual class. If tasks are correlated, the matrix M can be decomposed into a common $M_{0}$ and individual task‐specific $M_{t}$ parts, as

(2) $$d_{t}\left(x_{i},x_{j}\right)=\sqrt{{\left(x_{i}-x_{j}\right)}^{T}\left(M_{0}+M_{t}\right)\left(x_{i}-x_{j}\right)}$$

where $M_{0}$ and $M_{1},{\cdots ,M}_{T}$ are the global matrix and task‐specific additional matrices respectively. The larger the similarity is between the tasks, the larger the determinant of matrix $M_{0}$ relative to those of individual tasks $M_{t}$ . This idea was first applied to multi kNN by Parameswaran et al.^22^ Since that time many different algorithms have been developed for metric learning, as overviewed by Yang et al.^23^

**Figure 2:**
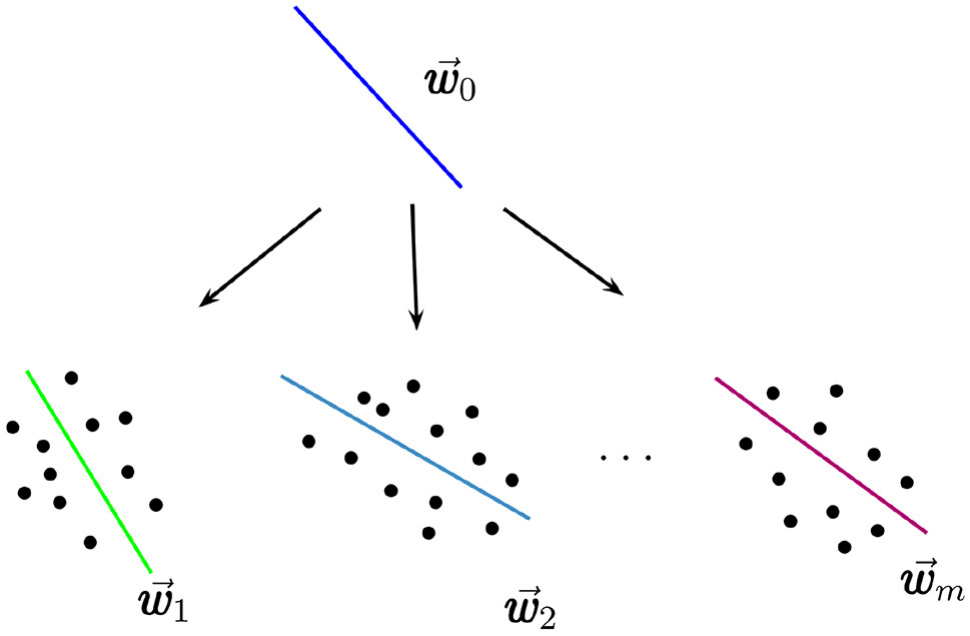
Multi‐task learning in Least Square Support Vector Regression (MLS‐SVR) identifies a common hyperplane $w_{0}$ , which carries the information of the commonality and $w_{i}=w_{0}+v_{i}$ , where the vector $v_{i}$ carries the information of the specialty. (Reprinted from *Pattern Recognition Letters, vol. 34*, Xu, S.; An, X.; Qiao, X.; Zhu, L.; Li, L., Multi‐output least‐squares support vector regression machines, Copyright (2013), with permission from Elsevier).

**Similarity learning**. Metric learning, in contrast to feature selection, directly optimises the parameters of the method itself. The main idea is that similar tasks can provide better generalization by using similar parameters. For example, when classifying several related properties one can identify a common separation hyperplane given by a vector *w_0_*, which will be only slightly different for separation hyperplanes *w_i_* for individual properties

(3) $$w_{i}=w_{0}+v_{i}$$

where $v_{i}$ accounts for features specific for property *i*. This separation is thus similar to that used for global and task‐specific matrices in eq. (2) where $w_{0}$ and $v_{i}$ correspond to matrices *M_0_* and *M_t_* respectively. Figure 2 exemplifies the intuition underlying this idea used to develop the Multi‐Task Least Square Support Vector Regression (MLS‐SVR) approach.^24^

**Figure 3:**
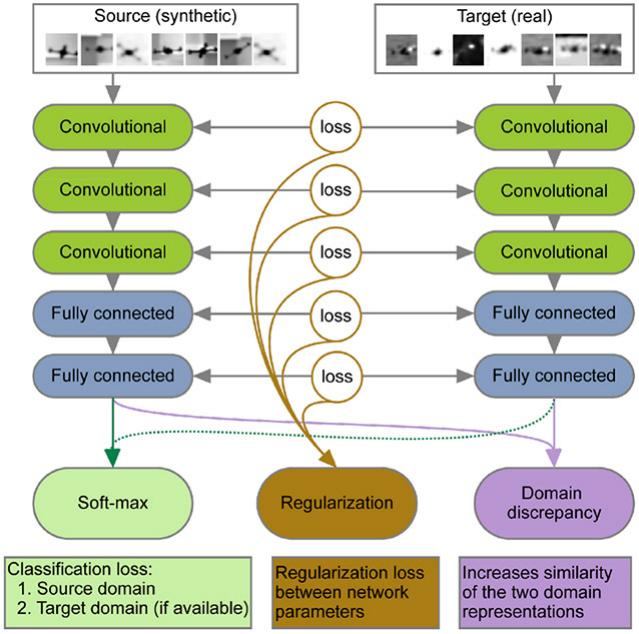
An example of neural network model using “soft parameter” sharing. Two networks are trained in parallel for each individual task. The soft parameter sharing is done by introducing a penalty function, which prevents neural network weights in both models from differing greatly, as well as by regularising neural network features at the last layer. Reprinted from ref. [25] under the Creative Commons license CC‐BY 4.0.

One of the promising current approaches in the field is based on MTL networks with “soft” parameter sharing (see Figure 3). The network facilitates multi‐task learning by regularising weights as well as features (which are defined as neural network activation patterns at the last layers) across the networks.^25^ The regularisation of weights corresponds to the sharing of model parameters while the regularisation of learning features across the last networks’ layers corresponds to feature regularisation. The algorithm can also be applied if no measurements are available for one of the tasks.

The information about task dependency can be used as *a priori* information and an example of multi‐task learning with the integration of taxonomy information has been presented by Rosenbaum et.^26^ The authors used a dataset of 112 human kinases extracted from ChEMBL. The Graph‐regularized multi‐task (GRMT) Support Vector Machine Regression and Top‐Down Multi‐task SVR were used to consider the relationship between these targets during modelling. The authors showed that hierarchical learning provided significantly better results compared to base models, as developed using STL approaches such as STL, or a model, which combined all data and ignored the kinases’ types.

Similarity learning is also a feature of Generative Topographic Mapping (GTM),^27^ which can be used both for visualization and molecular property prediction.^28^ GTM constructs a projection from a high‐dimensional descriptor space into a latent (usually 2D) space. Probabilities of the latent representations of molecules can be regarded as GTM descriptors and be used to build classification or regression models. Gaspar et al.^29^ proposed the Stargate GTM method, which projects both descriptors and multi‐target activity spaces into corresponding latent spaces and iteratively optimizes the joint probability distribution between the two mappings. The authors compared the method on data extracted from ChEMBL and showed that the Stargate GTM slightly outperformed conventional GTM but had a lower accuracy than Random Forest. It was also stressed that the model can act as a “gate”, which both predicts the activity profiles for a compound and finds areas in a descriptor space that are likely to have the desired activity profile. The latest feature is a particular advantage of Stargate GTM.

In machine learning there are a number of other approaches that can explore task similarity, including task clustering or multi‐level approaches as reviewed elsewhere.^9^

## 4 Simultaneous Feature and Task Similarity Learning

As it was aforementioned, networks with soft parameter sharing can provide a richer variety of network architectures (for review see^13^). Such networks can be used to simultaneously provide feature selection and task similarity learning. Let us show how this method could potentially be used to address domain adaptation. This problem is well known in the chemical industry and has been deeply studied by Sheridan,^30^ who demonstrated a loss of prediction accuracy in models for prospective validation of compounds, due to a time shift in chemical diversity. The problem of prospective validation can be easily cast to the multi‐learning domain by considering two tasks (prediction of past and new data, for which one may have just a few measurements) as two separate tasks.

## 5 Implementations of Multi‐Task Learning Approaches

Multiple software packages exist and are available in the computer science field, which provide tools for multi‐learning. As a rule, many articles are published by the authors together with their source code, which is frequently deposited on online repositories such as GitHub, allowing wide and immediate dissemination of information. The use of these software tools in chemoinformatics is not necessarily straightforward due to the need to make an interface with chemical structures. However, several efforts to port these packages to chemoinformatics are currently on‐going. In Table 1 we review several complete packages, which were developed to bring multi‐learning approaches to analyse chemical structures.

**Table 1:** “Chemistry aware” multi‐task learning approaches.

Package	Examples of supported algorithms	Availability
Chainer Chemistry	NFP, GGNN, RSGCN, WeaveNet, SchNet	https://github.com/ pfnet‐research
DeepChem	DAG, NNF, MPNN, TEXTCNN, WEAVE, IRV	https://github.com/ deepchem
OCHEM	The methods from Chainer Chemistry, DEEPCHEM, DNN, MLS‐SVM as well as MTL by property encoding as descriptors and feature net	http://ochem.eu

Chainer Chemistry (ChemChainer) ports several neural network architectures, which were recently introduced to work with graphs, to chemical structures. DeepChem supports the majority of ChemChainer methods as well as providing several other approaches, some of which were originally developed by the authors of the toolbox. DeepChem also provides a port of machine learning methods from the Scikit‐learn python package. Since the latter methods support only single‐task learning, DeepChem uses an embedded wrapper to calculate models for each task, and provides a combined result of STL models in way similar to that of MTL, thus allowing an easy comparison of STL and MTL models. Thus, the user can apply both types of methods to datasets containing several properties using a similar interface. ChemChainer and DeepChem are based on Python and are built around Chainer and TensorFlow frameworks, respectively. Both packages use the RDkit library,^31^ which provides a framework to translate chemical structures to graphs and the required representation for both packages.

**Figure 4:**
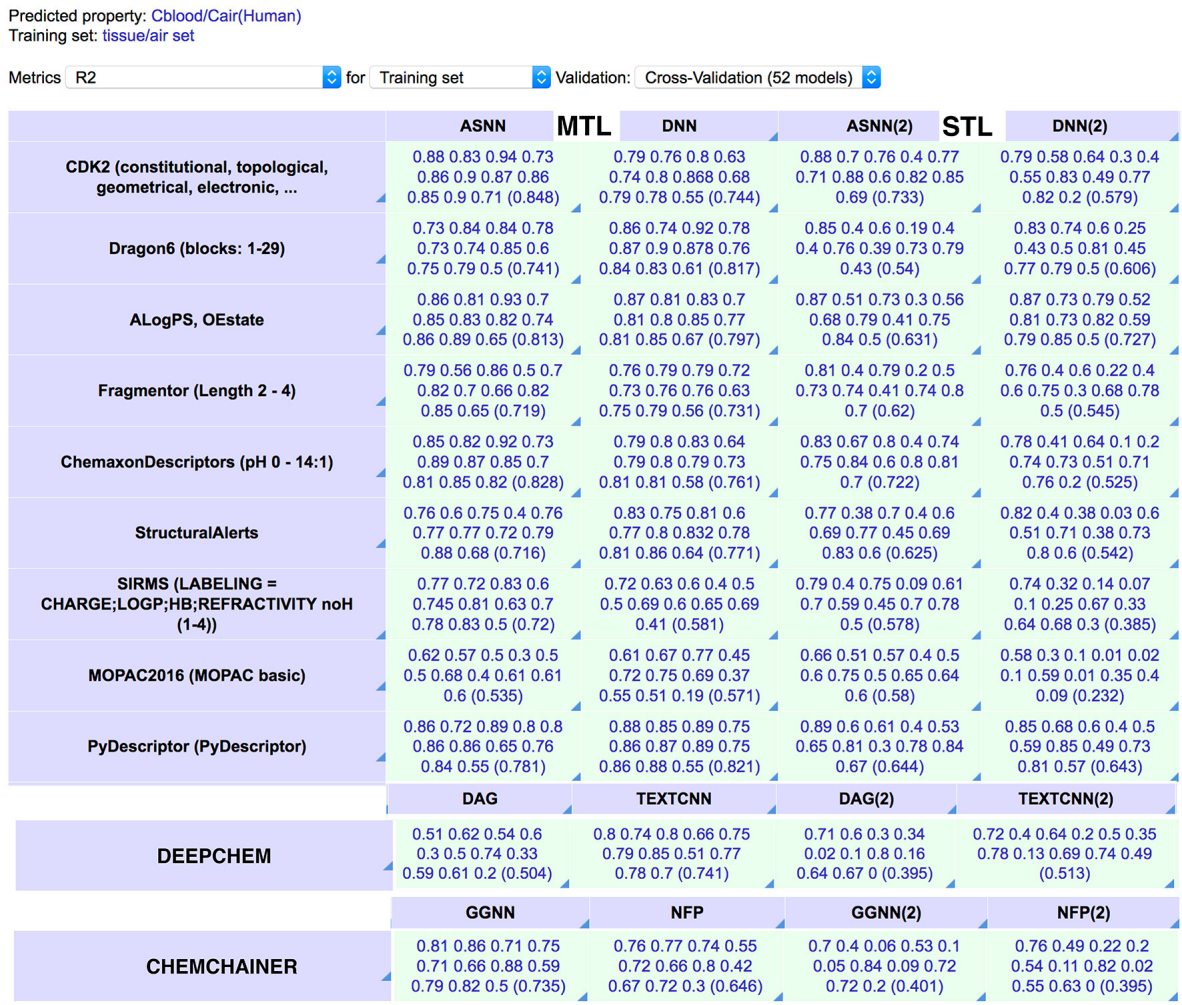
Example of MTL and STL using the comprehensive‐modelling view of the OCHEM platform. The RMSE of models on the left‐side columns (MTL) provide a higher squared correlation coefficient, R^2^, than models developed for each analysed property regardless of the descriptor set or method used. The models developed using DEEPCHEM and ChemChainer are based on chemical graphs. The values in parentheses indicate the average value or R^2^ for each analysis. ASNN – Associative Neural Networks;^6^ DNN – Deep Neural Network;^16^ DAG – Directed Acyclic Graphs;^43^ TEXTCNN – Text Convolutional Neural Network;^45^ NFP – Neural Network Fingerprint;^36^ GGCN – Gated Graph Neural Network.^41^

OCHEM provides^32^ a uniform interface to methods from both of these packages as well as several other methods supporting multi‐task learning, such as Associative Neural Networks, an implementation of Deep Neural Networks, a GPU implementation of Least Squares Support Vector Machines^33^ and several other approaches. An example of simultaneous prediction of tissue/air partitioning coefficients from Varnek et al.^8^ by different methods is shown in Figure 4.

Below we overview several methods implemented in these packages. The majority of these methods are neural networks that operate on chemical graphs. Thus, these approaches are different from traditional ones that analyse molecules by converting them to a set of descriptors. The first publication about the direct application of neural networks to graphs was proposed as an extension of recurrent neural networks in 2005.^34^ Interestingly, the first models based on chemical graphs were presented in the field of chemoinformatics about eight years earlier by Baskin et al.^35^

**Neural Network Fingerprints (NNF)**. The method shows that the representation of chemical structures as circular fingerprints (e. g. Morgan fingerprints or Extended Connectivity Circular Fingerprints (ECFP)) can be extended with a more advanced method based on neural networks.^36^

**Weave network**.^37^ This network was developed as an inspiration of convolutional neural networks. This network recreates atom and pair features on each layer based on the information in the previous layer, which resembles a weaving propagation of information through the network. The multiple layers (“weaves”) can be stacked to produce networks with more complex architectures.

**Renormalized Spectral Graph Convolutional Network (RSGCN)**.^38^ This network was developed to learn large graph‐structured networks, where the classification information is only available for a small number of samples but valuable additional information can be derived from the data graph structure of a much large number of unlabeled data points.

**A continuous‐filter convolutional neural network for modeling quantum interactions (SchNet)** 39 was developed to overcome the limitations of using grid‐based approaches, which work with discretized signals such as image pixels. The Comparative molecular field analysis (CoMFA)^40^ represents another example of a similar grid‐based approach coupled with PLS.

**Gated Graph Neural Network (GGNN)**. This network was specifically developed to predict sequences of outputs, allowing better predictions of their relationships.^41^ This algorithm was introduced by testing its performance on the bAbI suite tasks where it demonstrated a remarkable performance over existing algorithms. The bAbI tasks were specifically developed to test the reasoning capabilities of artificial intelligence systems, such as Path Finding and Shortest Path Finding, or automatic program verification.

**Message Passing Neural Networks (MPNN)** 42 are a generalisation of neural network architectures, which operate on graphs and update their node states using message passing. Examples of such networks are the NNF, GGNN, Weave and RSGCN networks considered above. The developed network was based on the GGNN architecture and had several improvements to decrease the computational cost and increase performance, e. g. optimisation of the final layers of the network (readout function which maps the whole graph to a feature vector), improvement of the scalability of training, etc. This allowed the authors to achieve superior results for 13 targets when co‐modelling electronic and energetic properties of molecules.

**Directed Acyclic Graphs (DAG)** 43 (or DAG Recursive Neural Network) consider molecules as directed graphs by iteratively taking each atom as a central one and defining the directions of all other bonds as outgoing from the central atom. The algorithm uses the atoms and their atomic features to propagate information through the graph to calculate properties. This operation is repeated for all atoms in a molecule and the result is used to train a neural network.

**Influence Relevance Voters (IRV)** 44 is a variation of a metric‐learning algorithm applied to molecular graphs. The motivation of this algorithm was to simulate the ability of humans to learn using just few examples or in a limit with a single example.

**Text Convolutional Neural Networks (TEXTCNN)** 45 uses neural network vectors trained on billions of words from Google News. These pre‐trained vectors serve as “universal” feature extractors that can be used to achieve excellent results for various problems. The method was adapted to work with SMILES by the developers of DEEPCHEM.

The variety of powerful and freely accessible methods will enable their wide use to address various multi‐learning tasks.

## 6 Open Issues

Despite the promising performance of MTL there are several issues, which either have not been properly addressed or remain open. Surprisingly, there is no good understanding as to which tasks are considered similar and could thus profit from multi‐learning.^13^,^46^, ^47^, ^48^ The main outstanding issue being that some tasks help each other and some do not; some compete for network capacity so that training them together actually worsens performance. Chen et al.^47^ stressed that, in general, multi‐learning neural networks can be rather hard to train because different tasks bring imbalances in the gradient calculations. The authors proposed an adaptive algorithm to estimate the weights of tasks dynamically during the training to improve prediction accuracy. Much remains to be explored in the design of neural network architectures, especially in the area of DNNs. A recent publication by Sturm et al.^49^ analysing the performance of DNNs on the ExCAPE‐DB of 70 million SAR datapoints, demonstrated a large dependency of the performance upon the hyperparameter choices. Optimising such parameters can be a costly operation, so determining general guidelines for estimating initial settings should be a point of future investigation. However, one can also formulate an even broader question: “Can we derive non‐linear dependences between tasks from data and use them to improve multi‐task learning?” Zamir et al. (a best paper award at the CVPR2018 conference)^48^ provided a method for automatic creation of taxonomy graphs for tasks. This approach has great prospects in chemoinformatics, e. g., for deriving and using the taxonomy of protein targets, viruses, toxicity endpoints, etc. in a fully data‐driven mode.

## 7 Summary

The multi‐task learning approaches are gaining popularity in various fields of science, including chemoinformatics. Successful use of these methods can result in models with higher prediction accuracies compared to the development of models for each individual property. The conditions when MTL can provide better results over STL are clearly formulated by Xu et al.^17^ As concluded by the authors MTL should be used for modelling correlated properties, but will degrade performance for uncorrelated properties. Structurally dissimilar molecules have no influence on the predictive performance of MTL, regardless of whether or not tasks are correlated. While these recommendations were for deep neural networks, they are likely to be valid for other multi‐learning approaches too and should be considered before deciding whether an MTL method can be employed. Finally, the development of a single MTL model is much faster and such a model occupies less memory and disk space compared to multiple single task models. This feature becomes important when increasing the number of simultaneously analysed properties. Examples of data sets that could potentially benefit from transfer learning and MTL with regards to QSAR modelling are given by Simoes et al.^50^ and include a) similar compounds measured under different experimental conditions; b) antimicrobial activities against genetically similar microorganisms; c) compounds with the same mechanism of action in homologous targets and high degrees of similarity in the binding pocket; d) non‐specific endpoints such as toxicity. When the endpoint has been measured exactly, but under different conditions or on e. g. different but correlated target organisms, one can also encode conditions as input descriptors. The availability of tools to perform multi‐learning is important for the widespread adoption and use of these methods by the scientific community.

## 8 Outlook

Both industrial and academic partners share high expectations from “Big Data” in chemistry, which is a new emerging area of research on the borders of several disciplines.^1^ Transductive learning in general, as well as multi‐learning approaches, will help to fully exploit the potential of such data by contributing models with higher prediction ability and coverage. These approaches will be important within the new federated learning project, a call for which was recently launched by the IMI. The future developments in this area should accommodate different data precision and accuracy from different sources, unbalanced datasets as well as sound calculation of the applicability domain and accuracy of predictions of multi‐models, which will be important for the use of these methods. Moreover, MTL can be combined with other types of networks, such as Recurrent Neural Networks (RNNs), to automatically design new chemicals with desired predicted properties.^51^

## Conflict of Interests

IVT is CEO of BIGCHEM GmbH, which licenses the OCHEM.^32^ The other authors declared that they have no actual or potential conflicts of interests.

## Biographical Information

*Sergey Sosnin is a Ph.D. student in the Center for Computational and Data‐Intensive Science and Engineering in Skolkovo Institute of Science and Technology. He gained his Master′s degree in Bioorganic chemistry from Moscow State University. His main research interests are chemoinformatics, computational methods of drug discovery, QSAR/QSPR*.

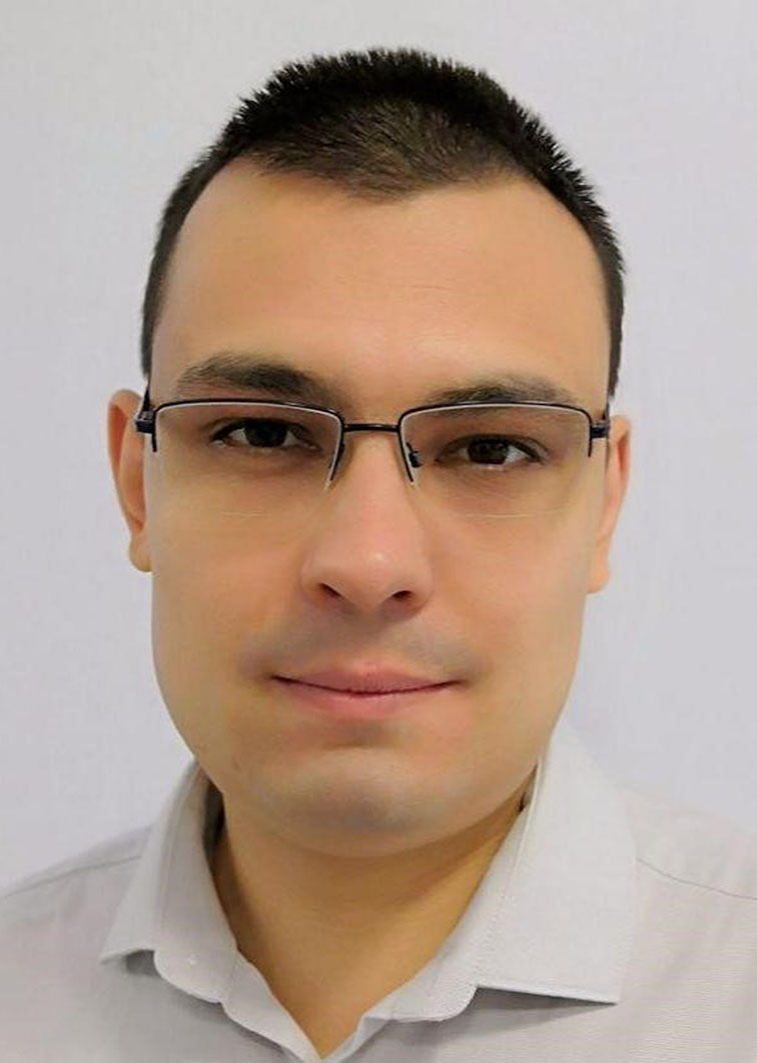

## Biographical Information

*Pavel Karpov obtained his PhD in organic and medicinal chemistry from Moscow State University, Russia. His main research interests lie in the development of new machine‐learning approaches for drug discovery. Now he is working as a postdoctoral fellowship in the Institute of Structural Biology in Helmholtz‐Zentrum Munchen*.

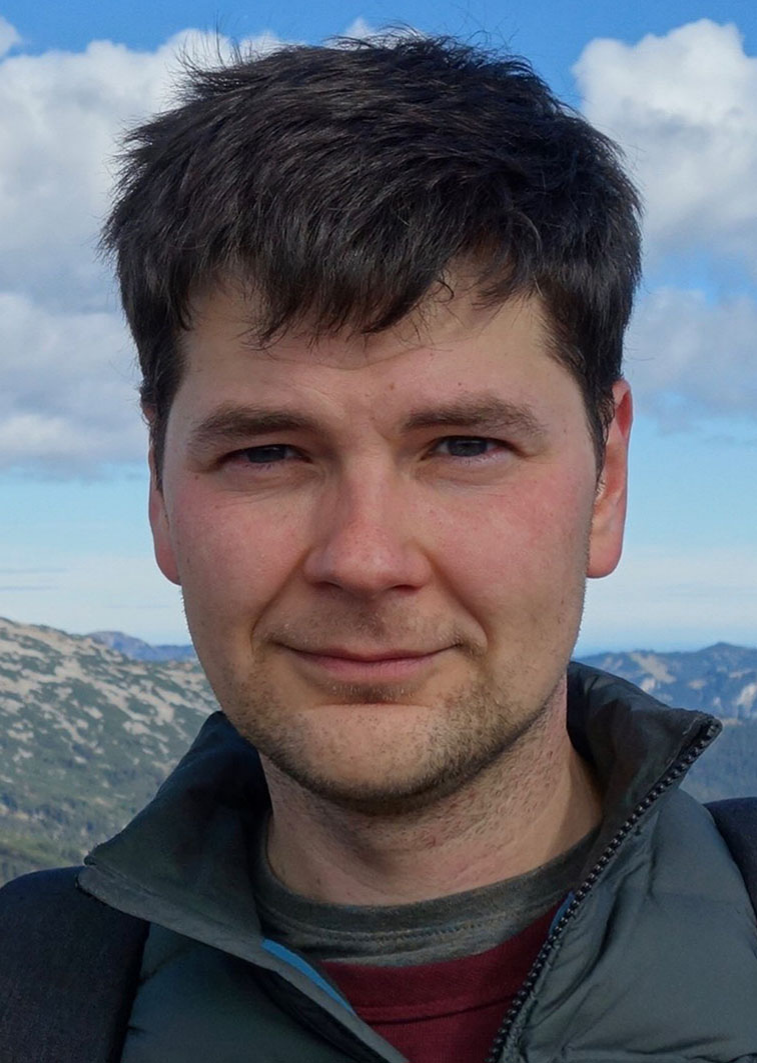

## Biographical Information

*Michael Withnall is a doctoral student at the Technical University of Munich, studying machine learning in chemoinformatics, and is industrially partnered with AstraZeneca. He has a Master′s degree in Chemistry from the University of Nottingham where he worked on OF‐DFT. His main research interests are chemoinformatics, quantum chemistry, and deep learning*.

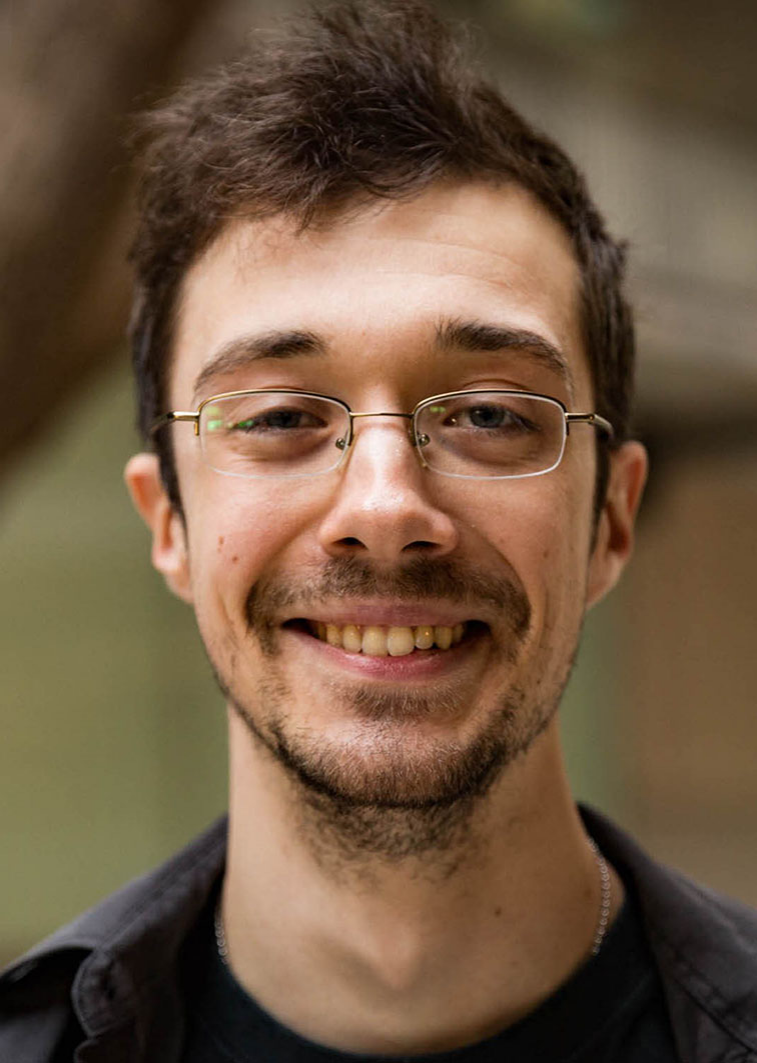

## Biographical Information

*Mariia Vashurina is a junior researcher at the Research Institute of Agricultural Microbiology of Russian Academy of Sciences. She was working under supervision of Dr. Tetko in Helmholtz Zentrum München and obtained her Master′s degree in Chemoinformatics from both Strasbourg University (France) and ITMO University (Russia). Currently she is working on the transmembrane molecular modeling and has a strong interest in machine learning methods*.

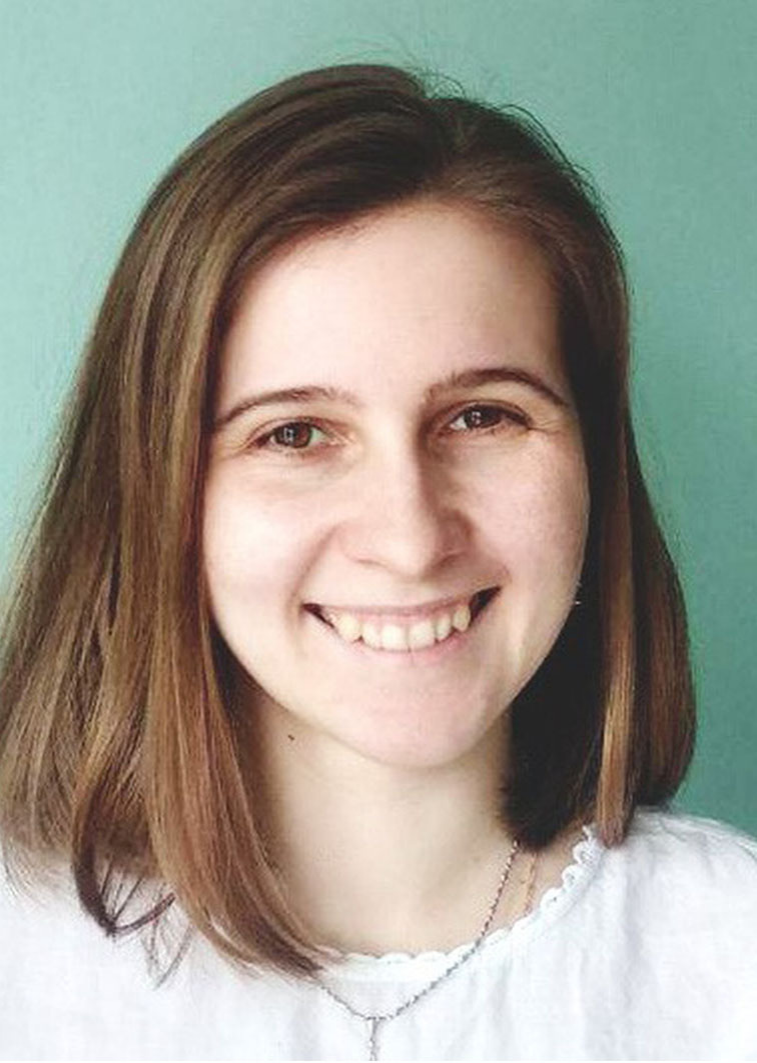

## Biographical Information

*Professor Maxim Fedorov is Director of the Centre for Computational and Data‐Intensive Science and Engineering (CDISE) at Skolkovo Institute of Science and Technology. He holds a PhD degree in Biophysics and DSc in Physical Chemistry (both degrees from the Russian Academy of Sciences). His current research is mainly focused on applications of artificial intelligence, data analysis and high‐performance computing in molecular sciences and biomedicine*.

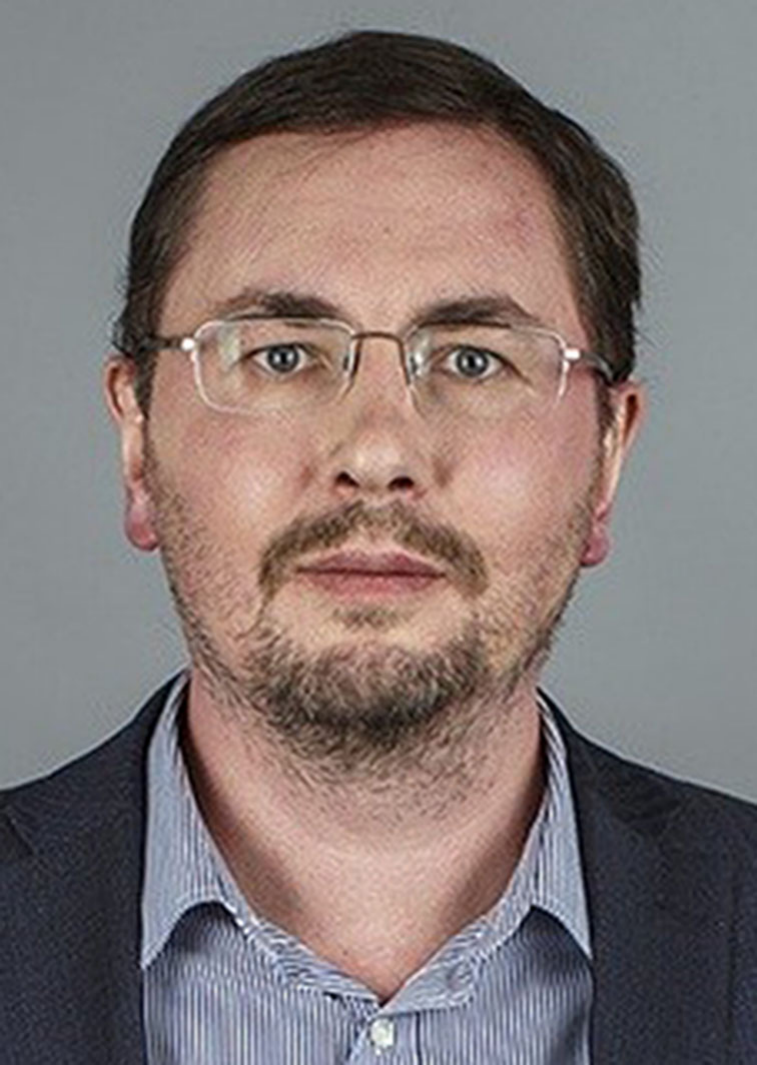

## Biographical Information

*Igor Tetko is Coordinator of Marie Skłodowska‐Curie Innovative Training Network European Industrial Doctorate Horizon2020 project “Big Data in Chemistry” and CEO of BIGCHEM GmbH (http://bigchem.de), which offers innovative solutions for Big Data analysis. His research interests include (Q)SAR/QSPR, development and application of machine learning approaches to predict physico‐chemical properties and biological activities of molecules*.